# Use or Misuse of Albumin in Critical Ill Patients

**DOI:** 10.3390/diseases11020068

**Published:** 2023-04-28

**Authors:** Fuat Hakan Saner, Bjoern-Ole Stueben, Dieter Peter Hoyer, Dieter Clemens Broering, Dmitri Bezinover

**Affiliations:** 1 Adult Transplant ICU, Organ Transplant Center of Excellence, King Faisal Specialist Hospital and Research Center, P.O. Box 3354, Riyadh 11211, Saudi Arabia; dbroering@kfshrc.edu.sa; 2 Department of General-, Visceral-, and Transplant Surgery, Medical Center University Duisburg-Essen, 45147 Essen, Germany; stuebenb@me.com (B.-O.S.); dieter.hoyer@uk-essen.de (D.P.H.); 3 Department of Anesthesiology and Critical Care 3400 Spruce Street, Hospital of the University of Pennsylvania, Philadelphia, PA 19104, USA; dbezinover@gmail.com

**Keywords:** immunology, volume treatment, sepsis, cirrhosis, hemodynamic

## Abstract

Since 1940 albumin has been used worldwide and is widely available commercially since this time. However, a meta-analysis in 1998 challenged the use of albumin and identified a trend toward higher mortality in critically ill patients who had received albumin. Since then, many studies including multicenter randomized controlled trials have been carried out investigating the safety and efficacy of albumin treatment in different patient cohorts. In this context, patient cohorts that benefit from albumin were identified. However, particularly in non-liver patients, the use of albumin remains controversial. In our comprehensive review, we would like to highlight the most important studies in the recent 20 years and therefore offer an evidence-based outlook for the use of albumin for patients treated in the ICU.

## 1. Introduction

The word albumin evolved from the old German word for protein, Albumen.

For over 30 years there has been an ongoing discussion on whether albumin should be given to critically ill patients. After more than 58 years of use of albumin a meta-analysis in 1998 [1] challenged this question. The authors reported a trend of higher mortality in patients who received albumin during their ICU stay. Although the difference in mortality was not significant (*p* = 0.06), this study attracted a lot of attention, which prompted a significant decrease in albumin use in the critical care setting.

After 3 years another meta-analysis showed that albumin could be used safely without harming the patient [2].

Albumin has different physiological functions. One of the most important is the maintenance of the colloid-osmotic pressure (COP), transport of hormones and drugs, as well as regulation of the acid-base balance and some immunologic effects [3].

It has been shown that low levels of albumin are associated with higher mortality [4]. Albumin replacement, therefore, seems reasonable in critically ill patients with hypoalbuminemia. One disadvantage of albumin is the high treatment cost. Randomized-controlled trials (RCT) showing any kind of benefit for albumin in critical care are still lacking. For that reason, the routine administration of albumin in this patient cohort is not recommended in the surviving sepsis campaign [5]. However, there is some evidence supporting the use of albumin particularly in patients with end-stage liver disease. This review aimed to highlight when to use or not use albumin in critically ill patients based on the best available evidence.

### 1.1. Historical Background

In 1901 Karl Landsteiner, an Austrian physician, discovered that the blood of two people agglutinates if mixed. He found that this effect was due to the contact of erythrocytes with blood serum. Landsteiner identified the three blood groups A, B, and O we know today. He later demonstrated that a blood transfusion between persons with the same blood group did not lead to hemolysis, while hemolysis did occur when the blood of persons of different blood groups was mixed [1].

Six years later in 1907, the first blood transfusion following a cross-match was carried out in New York Mount Sinai Hospital.

Albumin was one of the first products extracted from blood plasma. The first purified albumin for clinical use as a blood substitute was prepared in 1940 by Joseph Cohn, a chemist at Harvard medical school [2].

The first successful clinical use of albumin can be dated back to 1941, when, during the Pearl Harbor attack, albumin was used for multi-trauma and burn patients [3]. This led to an initiation of a so-called “albumin program”, facilitating albumin’s regular use in military to civilian hospitals [4].

### 1.2. Physiological Effects

**Figure 1:**
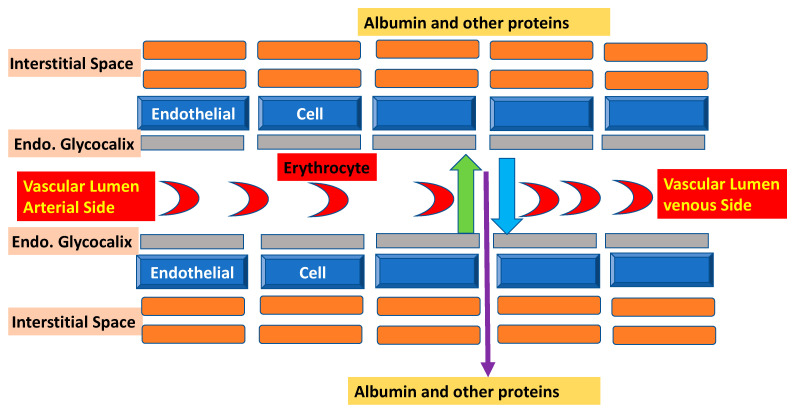
Interaction between endothelial cells, glycocalyx, albumin, and the interstitial space. The current understanding of vascular barrier function in the high-pressure segment of the vascular system includes an intact glycocalyx. Intact glycocalyx barrier competence is preserved even at albumin concentrations < 10 g/L. The green arrow shows the colloid oncotic pressure keeping fluid in the vascular lumen, while the blue arrow demonstrates the driving pressure out of the vessel. The thin purple arrow shows the transcapillary fluid flow, which results from a complex interaction of endothelial cells, glycocalyx, and albumin.

Albumin is a natural protein that is produced exclusively in the liver in amounts as high as 9–14 g/day. The median half-life is 18–19 days [3]. Albumin is responsible for maintaining the fluid balance between the intra- and extracellular space, accounting for about 90% of colloid osmotic pressure (COP) [5]. In critically ill patients, the interaction between COP and albumin is complex and is significantly affected by increased capillary permeability and precapillary escape [6]. Several experimental studies have demonstrated that albumin metabolism is more complex than the traditional understanding described by Starling as a simple inward oncotic gradient between a protein-rich intravascular space and a protein-low interstitial space. The endothelium is also protein-rich with the glycocalyx being a small layer consisting of glycoproteins, proteoglycans, and glucosamines, positioned on the luminal side of the endothelium. The glycocalyx performs an important barrier function in blood vessels [7]. It has been demonstrated that even at albumin concentrations as low as 10 g/L, the glycocalyx is preserved and prevents fluid shift to interstitial space (see Figure 1).

Another important function of albumin is its ability to transport many medications, vitamins, and amino acids [4].

Some studies have demonstrated albumin’s immunological effects. O’Brien et al. evaluated the role of cyclooxygenase-derived eicosanoid prostaglandin E_2_ (PGE_2_) as an immunosuppressive agent in patients with liver cirrhosis [8]. This study identified a significant increase in the level of PGE_2_ in patients with decompensated cirrhosis. PGE_2_ impairs the innate immune response in attenuating the inflammatory reaction and inhibiting the phagocytosis activity of macrophages. An infusion of albumin decreased the level of PGE_2_, which may reduce the risk of infections in cirrhotic patients.

Bortoluzzi et al. conducted an experimental study in which cirrhosis was induced in rats by inhalational exposure to carbon tetrachloride (CCl_4_) [9]. After cirrhosis was established, cardiac contractility was impaired which was caused by elevated levels of inducible nitric-oxide synthetase (iNOS) and TNF—alpha levels. After albumin infusion, levels of iNOS and TNF—alpha returned to baseline and cardiac contractility recovered. The same effect could not be achieved with an infusion of artificial colloids.

### 1.3. Safety

Albumin had been used worldwide without any safety concerns until the late 90′s. In 1998, a meta-analysis from the Cochrane Group’s Albumin Reviewers reported a higher mortality in critically ill patients when albumin was used. Although the results were not significantly different between groups (*p* = 0.06), the authors concluded that the use of albumin should be critically appraised and that the use of albumin should be restricted for use in patients subpopulations where a clear benefit has been demonstrated in randomized control trials (RCT) [10]. Some of their results were challenged 3 years later when Wilkes et al. failed to demonstrate an association between albumin administration and increased mortality [11].

Two years later another metanalysis published by the group of Jean-Louis Vincent was able to show that a low albumin serum concentration was associated with poor outcomes. The authors suggested that well-designed new trials were needed to characterize the effects of albumin treatment in patients with a low serum albumin concentration [12].

To address safety concerns, the SAFE study was conducted. This study was performed in Australia and New Zealand and included 6997 critically ill patients [13]. Patients randomly received 4% albumin or sodium chloride 0.9%. There was no difference in the 28-day mortality rate, but in the secondary endpoints, albumin was superior to sodium chloride in terms of volume load. In the first 4 days of treatment, 40% more volume was infused in the sodium chloride group. However, in the subgroup analysis, it was shown that trauma patients in the albumin group had a trend toward higher mortality rates. This effect was re-evaluated and the post-hoc data analysis was published three years later [14]. It demonstrated that patients with traumatic brain injury had significantly higher mortality in the albumin group. These results were initially surprising. However, considering that 4% albumin solution is hypo-osmolar, it can cause an intracellular fluid shift accompanying an increase in intracranial pressure resulting in higher mortality.

There are still conflicting recommendations from different societies regarding albumin administration. The European Society of Intensive Medicine (ESICM) recommends avoiding the use of albumin in neurosurgery, while the Scandinavian guidelines still recommend the use of albumin (20%) in patients with severe traumatic brain injury [15].

In the past, it had been assumed that the volume expansion effect of 20% albumin is superior to 5% albumin. In order to clarify this hypothesis, the SWIPE study was conducted [16]. In this evaluation, only hemodynamically unstable patients were included and assigned to receive either 20% or 5% albumin for volume resuscitation. Patients with traumatic brain injury were not included in the study. To achieve the same hemodynamic effect, 930 mL of 5% albumin was infused compared to 354 mL 20% albumin (*p* = 0.01). The rate of ICU survival was 97.4% in the 20% group and 91.1 % in the 5% group (*p* = 0.02).

Overall, there is enough evidence to suggest that the administration of albumin is safe

There are two additional issues that should be considered:

1. Albumin administration is associated with a significant sodium load ranging between 100–160 mmol/L depending on the albumin concentration (see Table 1);

2. 20% albumin seems to be the safest preparation while the hypo-oncotic 4% preparation should be avoided, particularly in patients with traumatic brain injury.

### 1.4. Hypoalbuminemia

Hypoalbuminemia (generally defined as serum albumin concentration < 30 g/L) is a very common condition in critically ill patients [17]. Hypoalbuminemia leads to increased capillary permeability with subsequent fluid redistribution from the intravascular to the interstitial space [18].

Hypoalbuminemia, regardless of the underlying mechanism, is associated with increased morbidity and mortality [13,19]. A meta-analysis examining hypoalbuminemia as a prognostic marker across 90 studies including critically ill patients found that for every 10 g/L decrease in albumin serum concentration, the OR of mortality increased by 137%, the risk of morbidity increased by 89%, and the length of hospital stay increased by 71% [13].

There is a clear correlation between albumin serum concentration and morbidity rates, however, the question remains if albumin replacement improves outcomes or if hypoalbuminemia is just a marker for severity of illness. At this time there is no clear evidence to support albumin replacement in critically ill patients with low serum albumin levels.

### 1.5. Albumin Use in Sepsis

The SAFE study indicated that patients with sepsis may benefit from albumin replacement [20]. In septic patients, hemodynamic goals were achieved with less volume replacement when albumin was used compared to crystalloids. The Albios study published in 2014 evaluated the effect of albumin in patients with sepsis and septic shock [21]. After randomization, patients in the albumin group received albumin with a target serum level of 3 g/dL. This procedure continued for the next 4 weeks or till discharge, which comes first.

After 28 days and 90 days, there was no difference in term mortality (31.8% vs. 32%; 41.1% vs. 43.6% after 90 days [21]. However, patients with septic shock demonstrated a significantly better outcome when albumin was used (RR = 0.87, 95% CI = 0.77–0.99).

The EARSS-albumin resuscitation study (EARSS Study) included only patients with septic shock. The authors were able to demonstrate improved survival in the group receiving albumin compared to the group receiving crystalloids. However, due to insufficient patient recruitment, the study was stopped before a clear conclusion could be drawn and was only published as an abstract [22]. A pooled analysis of all these 3 studies (SAFE, ALBIOS, EARSS studies) identified improved outcomes for patients resuscitated with albumin [23]. There is an ongoing RCT in Germany: “Albumin-replacement-in-septic-shock (ARISS)”. This study is currently recruiting patients with septic shock [24]. International guidelines for the management of sepsis and septic shock: Surviving Sepsis Campaign” graded recommendations for albumin administration in adults with sepsis or septic shock as weak [25].

### 1.6. Albumin and ARDS

As in septic patients, hypoalbuminemia in the setting of acute respiratory distress syndrome (ARDS) is associated with inferior outcomes [26]. Restricted fluid management with reduced fluid intake, use of furosemide, and albumin administration to achieve a negative fluid balance has been shown to be associated with improved oxygenation and reduced ventilation requirement in patients with ARDS. Unfortunately, restricted fluid management did not affect patient outcomes [27]. In an RCT including 40 patients with acute lung injury (ALI), patients were assigned to receive either albumin 20% plus furosemide or furosemide alone. The addition of albumin to furosemide results in an improvement of the Horovitz-Index, better hemodynamic stability, and net negative fluid balance [28].

Another metanalysis of three RCTs included 206 patients receiving either albumin or saline [29]. There was no significant difference in oxygenation and all-cause death between albumin and crystalloids groups

### 1.7. Albumin Replacement in Cirrhotic Patients

In patients with end-stage liver disease (ESLD), albumin is often used as both medication and/or as volume replacement. Most supportive evidence for the use of albumin in patients with ESLD is available for fluid replacement after large-volume paracentesis (>5 L ascites), for patients with hepatorenal syndrome (HRS), and for spontaneous bacterial peritonitis (SBP).

#### 1.7.1. Large-Volume-Paracentesis

Repeated large-volume paracentesis in patients with tense ascites is a well-recognized first-line treatment modality [30,31]. Draining a large volume of ascites (>5 L) may be associated with hemodynamic instability [32]. In order to prevent severe hypotension, it has been recommended to replace 8 g of Albumin for every liter of ascites [33,34]. In patients who are hemodynamically unstable or suffer from acute kidney injury (AKI), 8 g of albumin should be replaced for every liter of ascites, even if the amount of paracentesis is under 5 L. This is because the kidneys require stable hemodynamics to preserve their function [31,35]. Long-term albumin replacement in patients with cirrhosis should be recognized more as medical therapy rather than as volume replacement. In the ANSWER trial, 440 patients with cirrhosis and uncomplicated ascites who were treated with anti-aldosteronic drugs (≥200 mg/day) and furosemide (≥25 mg/day); they were randomly assigned to receive either standard medical treatment (SMT) or SMT plus human albumin (40 g twice weekly for 2 weeks, and then 40 g weekly) for up to 18 months. [36].

Thirty-eight of two-hundred and eighteen patients died with standard medical treatment (SMT) plus human albumin (HA), and 46 of 213 were in the SMT-only group. Overall 18-month survival was significantly higher in the albumin group (77% vs. 66%; *p* = 0.028). The data of this study indicate that albumin has beside volume effect and disease and immunology modifying effect. The MACHT trial (multicenter, randomized, double-blind, placebo-controlled), evaluated 196 liver transplant candidates on the waiting list [37]. Patients were randomly assigned to receive midodrine (15–30 mg/day) and albumin (40 g/15 days) and corresponding control patients placebos for one year. In contrast to the ANSWER trial, the authors did not find any difference in morbidity and mortality after 1 year.

The ATTIRE trial was a randomized, multicenter evaluation involving hospitalized patients with decompensated cirrhosis who had a serum albumin level of less than 30 g per liter at the time of enrollment [38]. Patients were randomly assigned to receive either a 20% human albumin solution for up to 14 days or until discharge, whichever came first, or standard care. A total of 777 patients underwent randomization.

Patients in the intervention group received a median of 200 g (25’th/75’th percentile 140–280 g), while the patients in the control group received a median of 20 g (0–120 g). Regarding kidney failure, infection, and hospital mortality there was no difference between both groups.

However, more serious adverse events occurred in the albumin group.

Although the study design in the MACHT and ATTIRE trials were not comparable, currently no recommendation for routine albumin replacement in hospitalized cirrhotic patients can be made [39].

#### 1.7.2. Spontaneous Bacterial Peritonitis

Spontaneous bacterial peritonitis (SBP) typically appears in patients with cirrhosis and ascites. Due to intestinal ischemia, the mucosal surface and intestinal epithelia are damaged, resulting in bacterial translocation to the ascites. These patients require antibiotic treatment, not surgical intervention.

In 1999, Sort et al. published a study regarding the use of albumin in the treatment of SBP [40]. In this study, 126 patients with cirrhosis and SBP were recruited either to receive cefotaxime (63 patients) or cefotaxime and albumin (63 patients). Cefotaxime was given daily, and albumin was given at a dose of 1.5 g per kilogram of body weight at the time of diagnosis, followed by 1 g per kilogram on day 3. Infection resolved in 94% of patients in the cefotaxime group (94%) and 98% in the albumin group (*p* = 0.36). However, acute kidney injury developed in 21 patients in the cefotaxime group, but only in 6 patients in the albumin group (10 %) (*p* = 0.002). Kidney dysfunction was associated with a significantly higher in-hospital mortality (29% vs. 10%, *p* = 0.01) and was also evident 3 months after the start of the study (41% vs. 22%, *p* = 0.03). It was also shown that in the non-albumin group, renin and aldosterone levels were significantly higher compared to the albumin group. This indicates that the non-albumin group was hypovolemic which resulted in impaired kidney perfusion and acute kidney injury.

Another systematic review was performed using MEDLINE and Embase databases to evaluate the effect of albumin in the setting of extraperitoneal infections [41]. Three RCTs comparing albumin and antibiotics to antibiotics alone in cirrhotic patients with extraperitoneal infections evaluated for mortality and renal dysfunction. There were no significant differences between groups regarding 30-day mortality or prevalence of renal dysfunction between groups.

#### 1.7.3. Hepatorenal Syndrome

Hepatorenal syndrome (HRS) is a serious complication in patients with cirrhosis and is associated with high morbidity and mortality. It is characterized by circulatory dysfunction that exceeds the compensatory mechanisms of the kidneys prompting vasoconstriction of the vas afferens resulting in decreased glomerular filtration (GFR). The definition of HRS has been challenged in the last 30 years. In 1996, the International Ascites Club first established a definition of HRS and has since modified it as new information has become available [42]. The diagnosis of HRS is based on the following parameters:

1. Presence of portal hypertension

2. Exclusion of any other reason for kidney impairment.

The current definition of HRS was published in 2015 by Angeli et al. [43]. The new version incorporated a new definition and classification based on the Kidney Disease Improving Global Outcome (KDIGO) criteria from 2012 (see Table 2) [44]. Stratification of HRS included 2 types. Type I (rapid reduction of renal function by doubling of initial serum creatinine to a concentration of at least 2.5 mg/dL) and type II (renal failure progression does not meet the criteria for type I) [42]. Type I has been replaced by HRS –AKI, and in HRS-NAKI, if AKI criteria are not fulfilled. HRS –NAKI is further stratified in HRS acute kidney disease (HRS-AKD) and HRS chronic kidney disease (HRS-CKD) if the time period exceeds 3 months.

Treatment of HRS-AKI should be performed with a vasopressor and albumin infusion. On day one, 1 g albumin per kg body weight should be infused followed by 20–40 g daily for 2–16 days [33].

A meta-analysis evaluated the effects of albumin and HRS Type 1 in 19 clinical studies which included 574 patients] [45]. The pooled percentage of patients achieving HRS reversal was 49.5% (95 % CI = 40.0–59.1%). Cumulative albumin dose increments of 100 g were accompanied by significantly increased survival (HR = 1.15; 95 % CI = 1.02–1.31; *p* = 0.023). Expected survival rates at 30 days among patients receiving cumulative albumin doses of 200, 400, and 600 g were 43.2 % (95 % CI = 36.4–51.3 %), 51.4 % (95 % CI = 46.3–57.1 %), and 59.0 % (95 % CI = 51.9–67.2), respectively.

This study suggests a dose-response relationship between infused albumin and survival in patients with HRS type 1.

In Table 3, the most important studies for cirrhotic patients are listed.

Based on 3 relevant studies, hydroxyethylstarch (HES) should be considered nephrotoxic [47,48,49]. Older studies have suggested that HES may cause renal dysfunction in kidney-transplant recipients. Legendre and colleagues have demonstrated osmotic nephrosis-like histological lesions in most transplanted kidneys after HES became the preferred plasma-volume expander for use in organ donors [50]. Similar histological lesions have been reported with other agents, including dextran, immunoglobulin, and mannitol [51,52]. This pathophysiology (including osmotic nephrosis) may also occur in patients treated with albumin. However, the SFAE study demonstrated that albumin administration did not result in this complication when used in critically ill patients [20].

Whether albumin has a protective effect in the context of AKI must be examined in further evaluations. Until then, its use cannot be generally recommended for use in patients with AKI.

### 1.8. Albumin and Renal Replacement Therapy

Some studies support the benefit of albumin administration during hemodialysis because of improved hemodynamic stability, improved fluid withdrawal, and increased overall safety [53,54].

Macedo et al. conducted a randomized “cross-over” study to evaluate this [53]. Patients were assigned to an albumin group, receiving 100 mL albumin (20% or 25%), or 100 mL NaCl 0.9%. The albumin group experienced fewer episodes of hypotension compared to the NaCl 0.9% group.

The RENAL study was a multicenter, prospective, randomized trial comparing two levels of intensity of continuous RRT in 1508 adult (>18 years) critically ill patients with AKI conducted in 35 ICUs in Australia and New Zealand [55,56]. Data from a subgroup of patients in this study underwent a post-hoc analysis to evaluate the effect of albumin 20 or 25% [57]. The authors found that albumin treatment was associated with more effective fluid removal. Studies with larger sample sizes confirming these data are still lacking.

### 1.9. Perioperative Albumin Replacement

Evidence supporting existing guidelines for perioperative fluid therapy is surprisingly weak [58]. The validity of Starling’s principle of microvascular fluid shifts has recently been challenged (see physiological effects). Norberg et al. conducted a prospective study comparing patients with esophageal and pancreatic surgery. Plasma albumin concentration was repeatedly measured over 72 h [59]. Plasma albumin concentration decreased rapidly from baseline (32.8 ± 4.8 g/L) until the start of surgical reconstruction (18.7 ± 4.8 g/L; *p* < 0.001). The overall decrease in albumin continued until 1 h after surgery falling to 40% of the initial value and was then stable for 72 h. Preexisting hypoalbuminemia would become more evident in this setting. Hypoalbuminemia has been shown to be associated with inferior outcomes in surgical patients [60]. Decisive data supporting albumin replacement in this clinical scenario is still lacking. There remains uncertainty if a low albumin plasma concentration itself is a biomarker for poor outcome and if albumin should be corrected to prior surgery.

In cardiac surgery, priming the heart-lung machine with albumin and crystalloid compared with crystalloid alone was associated with transient lower lactate levels and less fluid replacement [61]. The authors’ conclusion, however, was that crystalloid priming is safe in coronary artery bypass grafting surgery in adults.

Lee et al. evaluated in cardiac surgery patients the effect of albumin in a RCT with 200 patients. Patients with preoperative serum albumin <4 g/L received albumin as much as to achieve a serum level >4 g/L. [62]. The authors found that patients with serum albumin concentrations above 4 g/L had less risk of developing AKI.

Veno-arterial extracorporeal membrane oxygenation (VA-ECMO) improves perfusion in patients suffering from cardiogenic shock. ECMO therapy itself can trigger inflammation with capillary leak and intravascular volume depletion [63]. In a retrospective study of 196 patients undergoing VA-ECMO), patients were randomized to receive either balanced crystalloids or albumin with balanced crystalloids (1:2) (66).

The results of the study indicated a significantly better survival in the albumin before (38.4 vs. 25.7%, *p* = 0.026) and after propensity matching (43.9 vs. 27.6%, *p* = 0.025). The results were confirmed in multivariate regression analysis. Use of albumin improves hospital survival before (OR of 4.33 (95% CI = 2.01–9.33)and after propensity matching 3.1 (1.15–6.38).

## 2. Conclusions

Albumin administration is safe. Caution with the use of some concentrations of albumin solutions is warranted. Albumin 4% is hypo-osmolar and should generally be avoided, particularly in patients with intracranial pathology.

The highest-quality evidence exists for the use of albumin in patients with cirrhosis, particularly for the treatment of SBP, HRS, and large-volume paracentesis (>5 L). Patients with cirrhosis and hyponatremia scheduled for LT are at risk for osmotic demyelination syndrome postoperatively because of the rapid rise in serum sodium associated with the use of large volumes of albumin solutions. Albumin formulations with high sodium concentrations (and FFP transfusion Na = 170 mmol/L) should be avoided in this subgroup of patients.

For patients with sepsis or ARDS and undergoing ECMO, the evidence for albumin therapy is not robust enough to allow for a general recommendation. Albumin should be considered when hemodynamic stability cannot be achieved with crystalloids alone.

## Figures and Tables

**Table 1:** Most frequently used albumin solutions in Germany (no liability for completeness).

Name.	Concentration Albumin (g/L)	Sodium Content (mmol/L)	Company
Human Albumin “CSL Behring” 20% Infusionslösung	200	125	CSL Behring, Hattersheim, Germany
Alburex 5/20	50/200	140	CSL Behring, Hattersheim, Germany
Human Albumin “Octapharma” 25%—Infusionsflasche	250		Octapharma Pharmazeutika, Wien, Austria
Human Albumin Takeda 50 g/L; 200 g/L; 250 g/L Infusionslösung	50/200/250	130–160/100–130/130–160	Takeda Manufacturing Austria AG, Wien, Austria
Humanalbumin Kedrion 200 g/L Infusionslösung Humanalbumin Kedrion 250 g/L Infusionslösung	200 250	123–136 123–136	Kedrion SpA, Barga (LU), Italy
Humanalbumin Octapharma 50 g/L Infusionslösung Humanalbumin Octapharma 200 g/L Infusionslösung	50 200	143–157 143–157	Octapharma Pharmazeutika, Wien, Austria
Albutein 50 g/L/200 g/L	50/200	130–160	Grifols GmbH, Frankfurt, Germany
Plasbumin 20/25	200/250 g	145	Grifols GmbH, Frankfurt, Germany
Crealb 40 g/L; Crealb 200 g/L	40/200	140	Sanquin Plasma Products B.V., Amsterdam, The Netherlands

Source: Office for healthcare safety in Germany (Bundesamt fur Sicherheit im Gesundheitswesen) (14 March 2023): https://aspregister.basg.gv.at/aspregister/faces/aspregister.jspx;jsessionid=ROvgVvp6ibNQn7mKwgKJ0uV6-_J6QTAeGWHZIJZI4OpLGGl6IMnm!-345026110. Paul-Ehrlich Institut: https://www.pei.de/DE/arzneimittel/blutprodukte/albumine/albumine-node.html (accessed on 20 February 2023).

**Table 2:** Stages of acute kidney injury according to the International Club of Ascites [43].

Stage 1	Increase in serum creatinine ≥0.3 mg/dL (26.5 μmol/L) or increase in serum creatinine ≥1.5-fold to twofold from baseline
Stage 1a	Creatinine <1.5 mg/dL
Stage 1b	Creatinine ≥1.5 mg/dL
Stage 2	Increase in serum creatinine at least twofold to threefold from baseline
Stage 3	Increase in serum creatinine at least threefold from baseline or serum creatinine ≥4.0 mg/dL (353.6 μmol/L) with an acute increase ≥0.3 mg/dL (26.5 μmol/L) or the initiation of renal replacement therapy.

**Table 3:** Most important studies in cirrhotic patients and the use of albumin.

Study/Kind of Study/Author and Indication	Journal/Year	Number of Patients	Main Results
RCT/Sort et al. [40] SBP	NEJM/1999	126	Treatment of SBP with Albumin and antibiotics reduces significantly AKI and mortality
RCT/Sanyal et al. [46] HRS	Gastroenterology 2008	56	Terlipressin and Albumin are effective in the treatment of HRS
Meta-Analysis Bernardi et al. [35]/ Large-volume Paracentesis	Hepatology 2012	1225	Albumin significantly reduced hemodynamic instability after paracentesis
RCT/Answer Trial/Caraceni et al. [36] Long-term albumin replacement in outpatient clinic	Lancet 2018	431	Long-term albumin treatment (18 months) improved overall survival
RCT/ATTIRE Trial/China et al. [38] Albumin replacement in hospitalized patients with serum albumin <30 g/L	NEJM 2021	777	Short-term albumin replacement does not affect new infection, kidney dysfunction, or death 15 days after starting the treatment
RCT/MACHT Trial/Sola et al. [37] /Midodrine and albumin replacement for 1 year	J Hepatol 2018	196	Midodrine and albumin infusion did not improve survival after 1 year.

Abbreviations: SBP: spontaneous bacterial peritonitis. HRS: Hepatorenal syndrome. RCT: randomized controlled trial. AKI: Acute kidney injury. Albumin and Acute Kidney Injury.

## Data Availability

Review, no original data sheets are available.
